# Local Differences in the Toxin Amount and Composition of Tetrodotoxin and Related Compounds in Pufferfish (*Chelonodon patoca*) and Toxic Goby (*Yongeichthys criniger*) Juveniles

**DOI:** 10.3390/toxins14020150

**Published:** 2022-02-18

**Authors:** Masaaki Ito, Risako Furukawa, Shino Yasukawa, Masaya Sato, Hikaru Oyama, Taiki Okabe, Rei Suo, Haruo Sugita, Tomohiro Takatani, Osamu Arakawa, Masaatsu Adachi, Toshio Nishikawa, Shiro Itoi

**Affiliations:** 1 Department of Marine Science and Resources, Nihon University, Fujisawa 252-0880, Japan; brma21003@g.nihon-u.ac.jp (M.I.); brri17145@g.nihon-u.ac.jp (R.F.); brsh21020@g.nihon-u.ac.jp (S.Y.); brma20006@g.nihon-u.ac.jp (M.S.); brhi20501@g.nihon-u.ac.jp (H.O.); o.taiki.nihon.u@gmail.com (T.O.); suo.rei@nihon-u.ac.jp (R.S.); sugita.haruo@nihon-u.ac.jp (H.S.); 2 Graduate School of Fisheries and Environmental Sciences, Nagasaki University, Nagasaki 852-8521, Japan; taka@nagasaki-u.ac.jp (T.T.); arakawa@nagasaki-u.ac.jp (O.A.); 3 Graduate School of Pharmaceutical Sciences, Tohoku University, Aoba, Aramaki, Aoba-ku, Sendai 980-8578, Japan; masaatsu.adachi.d7@tohoku.ac.jp; 4 Laboratory of Organic Chemistry, Graduate School of Bioagricultural Sciences, Nagoya University, Chikusa, Nagoya 464-8601, Japan; nisikawa@agr.nagoya-u.ac.jp

**Keywords:** food web, *Planocera*, pufferfish, toxic flatworm, toxic goby, toxification

## Abstract

Tetrodotoxin (TTX)-bearing fish ingest TTX from their preys through the food chain and accumulate TTX in their bodies. Although a wide variety of TTX-bearing organisms have been reported, the missing link in the TTX supply chain has not been elucidated completely. Here, we investigated the composition of TTX and 5,6,11-trideoxyTTX in juveniles of the pufferfish, *Chelonodon patoca*, and toxic goby, *Yongeichthys criniger*, using LC–MS/MS, to resolve the missing link in the TTX supply chain. The TTX concentration varied among samples from different localities, sampling periods and fish species. In the samples from the same locality, the TTX concentration was significantly higher in the toxic goby juveniles than in the pufferfish juveniles. The concentration of TTX in all the pufferfish juveniles was significantly higher than that of 5,6,11-trideoxyTTX, whereas the compositional ratio of TTX and 5,6,11-trideoxyTTX in the goby was different among sampling localities. However, the TTX/5,6,11-trideoxyTTX ratio in the goby was not different among samples collected from the same locality at different periods. Based on a species-specific PCR, the detection rate of the toxic flatworm (*Planocera multitentaculata*)-specific sequence (cytochrome *c* oxidase subunit I) also varied between the intestinal contents of the pufferfish and toxic goby collected at different localities and periods. These results suggest that although the larvae of the toxic flatworm are likely to be responsible for the toxification of the pufferfish and toxic goby juveniles by TTX, these fish juveniles are also likely to feed on other TTX-bearing organisms depending on their habitat, and they also possess different accumulation mechanisms of TTX and 5,6,11-trideoxyTTX.

## 1. Introduction

Tetrodotoxin (TTX) is a popular potent neurotoxin known in Japan as a marine toxin that causes food poisoning, and the lethal dose in humans is estimated to be around 1 to 2 mg [1]. The name of TTX traces to the family Tetraodontidae [2], since it was first isolated from pufferfish. It was considered to be specific to pufferfish until 1964 when its molecular structure was independently reported by three research groups [3,4,5]. This led to the discovery that tarichatoxin of the Californian newt, *Taricha torosa*, was identical to TTX [6], and since then, the toxin has been detected in a very wide range of taxa [7,8,9]. TTX has also been detected in marine bacterial cultures, suggesting that the toxin is biosynthesized by bacteria and is accumulated in pufferfish through the food chain [7,8,9,10], and an enhanced TTX synthesis by TTX producer bacteria inside their hosts has also been hypothesized [8,11,12]. Indeed, it has been shown that pufferfish remain non-toxic when reared on non-toxic food but are toxified when they ingest TTX-containing feed [13,14]. These observations supported the speculation that TTX accumulated in pufferfish is from the food chain. On the other hand, some argue that the amount of TTX accumulated in the pufferfish cannot be accounted for by the amount of TTX obtained through the food chain, because marine bacterial cultures can only biosynthesize very small amounts of TTX [11,15,16,17].

There have been several attempts to resolve this apparent contradiction. For example, it was shown that the pufferfish, *Takifugu niphobles* (currently, *Takifugu alboplumbeus*) efficiently accumulates TTX by feeding on the eggs of another pufferfish, *Takifugu pardalis*, suggesting that TTX circulates among higher trophic predators, such as pufferfish species [14]. It was observed that *T. alboplumbeus* also ingests large amounts of TTX by feeding on the toxic ribbon worm *Cephalothrix simula* (Nemertea, Palaeonemertea) and the toxic flatworm *Planocera multitentaculata* (Platyhelminthes, Polycladida, and Acotylea) [18,19,20]. Interestingly, the larvae of *P. multitentaculata* and related species were also found to be involved in the toxification of various TTX-bearing fish juveniles, and an edible bivalve, *Azumapecten farreri akazara*. Flatworm larvae are considered to be important suppliers of the toxin to TTX-bearing organisms [21,22]. Although TTX-bearing flatworms appear to be largely restricted to the genus, *Planocera*, members of which are widely distributed in the waters around the Japanese archipelago [23,24,25,26,27], and other flatworms such as the cotylean flatworm, *Prosthiostomum trilineatum*, have also recently been discovered to contain the toxin [28].

These TTX-bearing flatworms have been found to harbor TTX and 5,6,11-trideoxy-TTX, which was comparable to the amount of TTX [28,29]. When TTX-bearing fish acquire the toxin from their prey, it would be expected that they would ingest it and its related compounds directly from their prey, and that the compositional ratios in the prey would be reflected in that of the predators. However, although differences in toxicity among regions have been reported for various fish species [30,31,32,33], there have been no reports comparing the composition of TTX and 5,6,11-trideoxyTTX among the populations from different localities.

**Figure 1:**
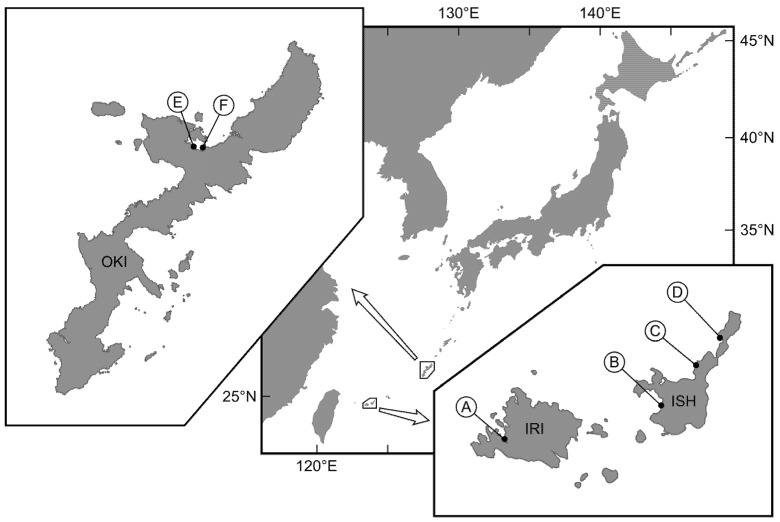
Sampling localities for the pufferfish, *Chelonodon patoca*, and the toxic goby, *Yongeichthys criniger*, used in this study. Sampling details can be seen in the inset, particularly regarding the three islands: “A” in Iriomote Island (IRI), “B–D” in Ishigaki Island (ISI), and “E” and “F” in Okinawa Island (OKI), Japan. The white arrows indicate the expansion of the small box on the main map.

Therefore, in this study, we investigated the compositional ratios of TTX and 5,6,11-trideoxyTTX in fish juveniles of the pufferfish, *Chelonodon patoca*, and the toxic goby, *Yongeichthys criniger*, and compared the composition of TTX and 5,6,11-trideoxyTTX in these species among different localities in the Ryukyu Islands (Figure 1) and across different months/years. Based on the results, we discuss the unidentified toxification process of these TTX-bearing fish species, and the difference in the TTX accumulation mechanisms between the two fish species, which requires clarification when considering the evolution of TTX-bearing organisms.

## 2. Results

### 2.1. Composition of TTX and 5,6,11-TrideoxyTTX in the Pufferfish

**Figure 2:**
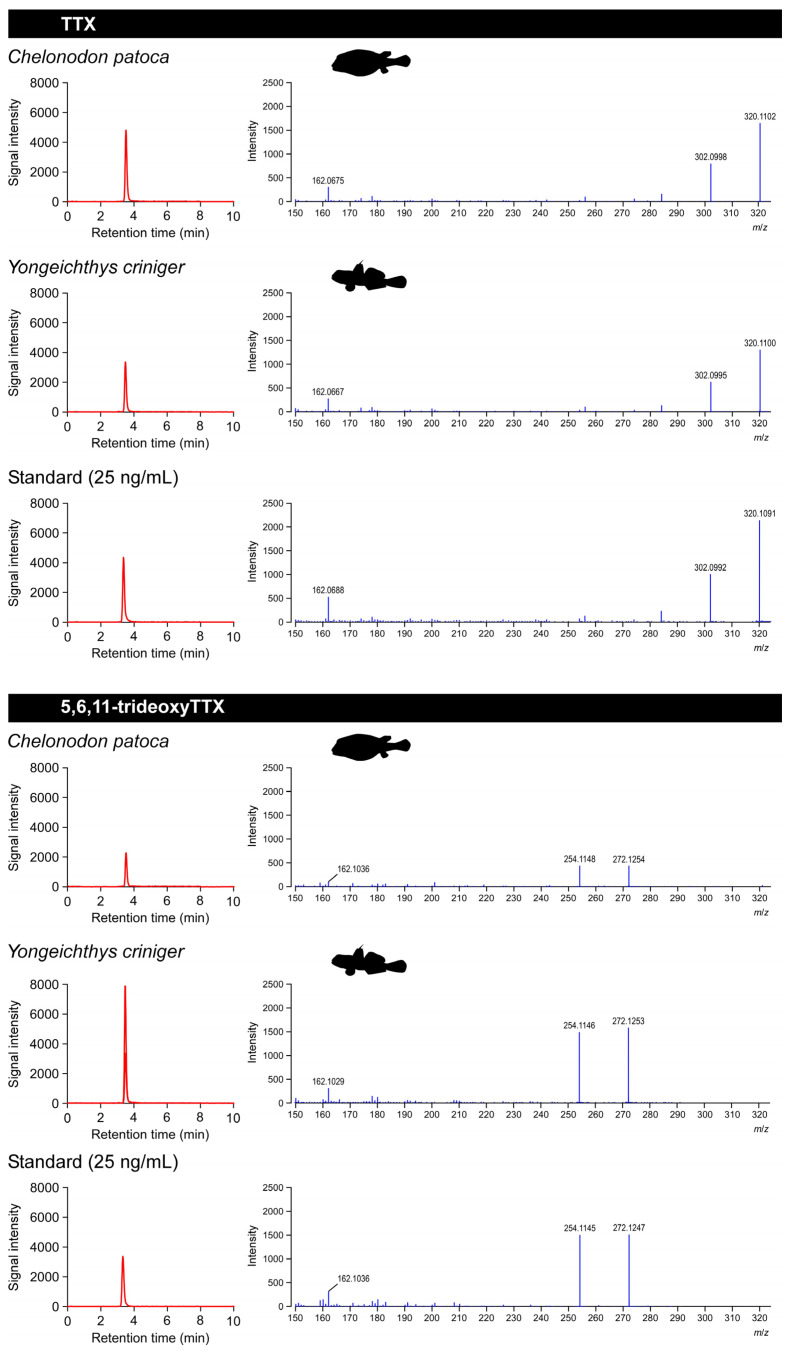
Sampling LC–MS/MS patterns of the TTX and 5,6,11-trideoxyTTX from the pufferfish, *Chelonodon patoca*, the toxic goby, *Yongeichthys criniger*, and 25 ng/mL standard. Left panels represent LC–MS/MS chromatograms, while the right panels represent the precursor and product ion mass spectra for TTX and 5,6,11-trideoxyTTX.

**Figure 3:**
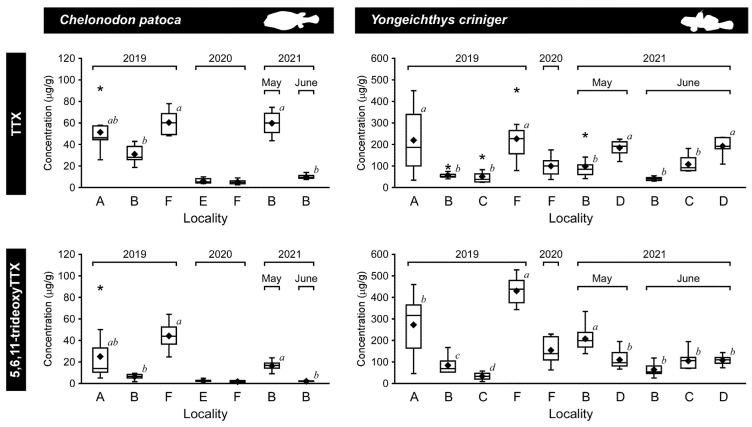
Box and whisker plot comparing the mean and median TTX and 5,6,11-trideoxyTTX concentrations (μg/g) for the pufferfish, *Chelonodon patoca*, and the toxic goby, *Yongeichthys criniger*, sampled from the southern islands (A, Iriomote; B–D, Ishigaki; E and F, Okinawa) of the Japanese Archipelago, in 2019–2021. The upper and lower edges of each box represent the value of the 3rd and 1st quartile, respectively, while the length of the whiskers reflects the variability outside these two quartiles. The line and diamond within the boxes represent the values of median (2nd quartile) and mean, respectively. Asterisks represent outliers. Letters “A–F” represent sampling localities corresponding to waters shown in Figure 1. Different alphabetical letters (*a*–*d*) within the box and whisker plot indicate a statistically significant difference among the measured values in the year (*a* > *b* > *c* > *d*, Tukey’s HSD post hoc test, *p* < 0.05), except for the pufferfish in 2020 and 2021 (Student’s *t*-test, *p* < 0.05).

TTX and 5,6,11-trideoxyTTX were detected in all individuals of the pufferfish, *C. patoca*, analyzed in this study (Figure 2, Table 1): concentrations of TTX and 5,6,11-trideoxyTTX ranged from 5.2 ± 2.0 to 60.4 ± 10.0 μg/g and 1.8 ± 0.8 to 37.0 ± 12.0 μg/g, respectively (Figure 3). In the pufferfish, *C. patoca*, the concentration of TTX in all the individuals analyzed were consistently 1.8 ± 0.6 to 5.6 ± 2.3-fold higher than that of 5,6,11-trideoxyTTX, while varying among localities and months/years. In 2019, all the pufferfish individuals were collected in June, when the TTX concentration was higher in the individuals from Iriomote Island (“A” in Figure 1) and Okinawa Island (“F” in Figure 1) than in those from Ishigaki Island (“B” in Figure 1). In 2020, pufferfish individuals were collected at two different localities of Okinawa Island in July. The TTX concentration in both populations (“E” and “F” in Figure 1) was lower than that in individuals collected in 2019. In 2021, the TTX concentration in the individuals from Nagura River (“B” in Figure 1) in Ishigaki Island was higher in May than in June, and these values in May and June were significantly higher and lower, respectively, than those in June 2019.

### 2.2. Composition of TTX and 5,6,11-TrideoxyTTX in the Toxic Goby

TTX and 5,6,11-trideoxyTTX were detected in all the sampled individuals of the toxic goby, *Y. criniger* (Figure 2, Table 1), with a concentration of TTX and 5,6,11-trideoxyTTX of 40.6 ± 7.6 to 226.5 ± 95.4 μg/g and 33.6 ± 14.6 to 427.0 ± 56.3 μg/g, respectively (Figure 3). In *Y. criniger*, the ratio of TTX/5,6,11-trideoxyTTX varied between 0.5 ± 0.2 and 1.9 ± 0.6 fold (Table 1): the ratio in all the individuals collected at estuarine waters in Fukido River (“C” in Figure 1) and coastal waters of Hirakubo (“D” in Figure 1) in Ishigaki Island was >1.0, while for those collected at Iriomote Island (“A” in Figure 1), Nagura River (Ishigaki Island, “B” in Figure 1) and Okinawa Island (“F” in Figure 1), it was < 1.0. A difference in the TTX concentration was also observed among populations from different localities. The individuals collected at Iriomote Island, Ishigaki Island (Hirakubo) and Okinawa Island showed higher TTX concentrations than those at other sampling localities.

### 2.3. Difference in the TTX Concentration of the Pufferfish and Toxic Goby

The TTX concentration between the simultaneously collected pufferfish and toxic goby was significantly different (Figure 3). In Irimote Island (“A” in Figure 1), the TTX concentration of the goby was significantly higher than that of the pufferfish in June 2019 (*p* < 0.05). Similarly, the TTX concentration of the goby from Ishigaki Island (“B” in Figure 1) and Okinawa Island (“F” in Figure 1) was also significantly higher than that of the pufferfish (*p* < 0.05).

### 2.4. Planocera multitentaculata-Specific Sequence (COI) from Intestinal Contents of TTX-Bearing Fish

#### 2.4.1. The Pufferfish, *Chelonodon patoca*

**Figure 4:**
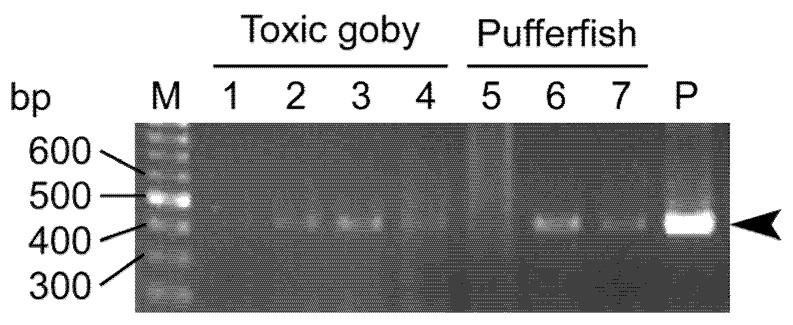
Typical electrophoretic patterns of *Planocera multitentaculata*-specific PCR products from the intestinal contents of the goby, *Yongeichthys criniger* (Lanes 1–4), and the pufferfish, *Chelonodon patoca* (Lanes 5–7). Arrows indicate PCR products specific to the sequence of *P. multitentaculata*. Lane M, molecular weight marker; P, positive control. Lanes 1–4 refer to the toxic goby samples collected at Okinawa Island (“F” in Figure 1), and lanes 5–7 to the pufferfish samples collected at Okinawa Island (“E” in Figure 1).

In June 2019, *P. multitentaculata*-specific sequences were detected in 10% of the *C. patoca* juveniles collected from Okinawa Island (“E” and “F” in Figure 1) but not in any from Iriomote Island (“A” in Figure 1) or Ishigaki Island (“B” in Figure 1) (Table 1; Figure 4). In the following year, the sequence was detected in as many as 70–90% of the pufferfish juveniles collected from two different localities in Okinawa Island in July 2020 (Table 1). The sequence was not detected in any of the pufferfish juveniles collected from Ishigaki Island in May and June 2021 either (Table 1).

#### 2.4.2. The Toxic Goby, *Yongeichthys criniger*

The detection rate of the *P. multitentaculata*-specific sequences varied among localities and months/years (Table 1). In June 2019, it was detected in 40–60% of *Y. criniger* individuals collected from Iriomote Island (“A” in Figure 1) and Ishigaki Island (“B” and “C” in Figure 1), although the sequence was not detected in those from Okinawa Island (“F” in Figure 1) (Table 1; Figure 4). While it was detected in 70% of the individuals collected from Okinawa Island in July 2020, it was not found in those from Ishigaki Island in May and June 2021 (Table 1).

## 3. Discussion

TTX-bearing organisms have been found in diverse taxa, and the search for the source of the toxin from the interaction of predators, such as pufferfish, and TTX-bearing prey has gradually been revealed [9,19,20,21,34]. TTX is thought to ultimately accumulate in higher trophic predators, such as pufferfish, through the food web, starting from bacteria [7,8]. On the other hand, the biosynthesis of TTX is understood to be different in TTX-bearing terrestrial animals [9,35,36,37], because the chemical profiles related to TTX are different between newts and pufferfish [38,39]. The toxic larvae of *P. multitentaculata* and related species are understood to contribute to the toxification of TTX-bearing fish juveniles, such as those of pufferfish (*T. alboplumbeus* and *C. patoca*) and the toxic goby, *Y. criniger*, as described above [19,21].

In the present study, although the sampling was only a snapshot, the detection rate of the toxic flatworm sequences varied by fish species, sampling period, and locality, and together with previously reported data [21], suggests that the juveniles of *Y. criniger* and *C. patoca* accumulated TTX partly from the larvae of the toxic flatworm, *P. multitentaculata*, and additionally from other TTX-bearing organisms. Furthermore, the amount of TTX differed significantly between *C. patoca* and *Y. criniger*, although they are sympatric [21] (in this study). The difference between the two species in TTX accumulation might be due to the ecological differences between two fish species. Juveniles of the pufferfish migrate between the sea and the river at the mouth of the estuary in accordance with the ebb and flow of the tide, whereas those of the toxic goby settle in a specific location. Therefore, while the pufferfish move with the surrounding environmental water and prey on food at the destination, the goby may feed on the food that comes in with the influx when the surrounding water around them is replaced. This behavior of the gobies is likely to give them a higher chance of encountering the toxic flatworm larvae, but they can only feed efficiently when a large number of prey larvae are adrift, and whether or not the larvae drift in is a matter of chance. The toxicity of the toxic goby juveniles differed considerably among localities, which is consistent with previous reports [21,30,33]. The accessibility of the toxic flatworm larvae in the goby’s habitat is thus a determinant of the extent of toxicity of the goby. However, this research has only attempted to detect flatworms, and cannot detect other TTX-bearing organisms even if they are fed. It would be necessary to investigate them comprehensively by NGS analysis.

The transferring of TTX from prey organisms has been reported in the pufferfish genus *Takifugu*, such as *T. rubripes* and *T. alboplumbeus* [13,14,19,40]. Alternatively, although the proposed composition of TTX and biosynthetic intermediates has been investigated in various species [41,42,43,44,45], there have been no studies on how these substances in the prey are ingested and transferred to predators. Therefore, it was speculated that various TTX-related compounds in the prey would be directly ingested and accumulated in the body of predators, such as ribbon worms and newts [46,47,48,49].

**Figure 5:**
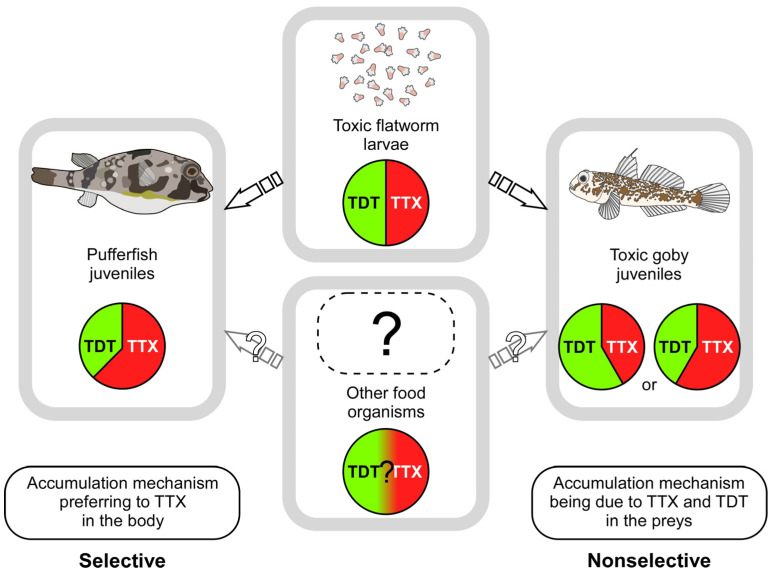
Differences in the TTX accumulation mechanisms between the pufferfish, *Chelonodon patoca*, and the toxic goby, *Yongeichthys criniger*. The pufferfish always showed higher concentrations of TTX than of 5,6,11-trideoxyTTX, whereas the ratio of TTX to 5,6,11-trideoxyTTX in the toxic goby varied depending on the location. The pufferfish appears to possess selective TTX accumulation mechanisms, while the toxic goby seems to rely on nonselective mechanisms. TTX-bearing flatworm species contain equal concentrations of TTX and 5,6,11-trideoxyTTX [28,29]. TDT, 5,6,11-trideoxy-TTX.

In this study, the accumulation of TTX and its related compound, 5,6,11-trideoxyTTX, in the juveniles of the pufferfish, *C. patoca*, and the toxic goby, *Y. criniger*, which are known to have high concentrations of TTX, was examined. The concentration of TTX was always two to five times higher than that of 5,6,11-trideoxyTTX in the pufferfish, although the concentrations of the TTX did vary. In contrast, the TTX/5,6,11-trideoxyTTX ratio in the toxic goby was different among sampling sites, although there was no difference in the ratio between the time periods. Additionally, the ratio was different between the pufferfish and goby collected from the same location, at the same time. These results suggest that the pufferfish and goby may ingest TTX by feeding on different prey, and that carriers and receptors involved in binding, transporting, and accumulating TTX and other compounds may have different binding capacities to TTX and 5,6,11-trideoxyTTX (Figure 5). TTX-binding protein found in the plasma is known to play a role in the transport of TTX in the body by binding to ingested TTX in puffer fish [50]. This TTX-binding protein has been reported to have a higher binding capacity to 5,6,11-trideoxyTTX than to TTX in the case of the pufferfish, *C. patoca*, and this binding capacity varies depending on the species of pufferfish [50], whereas there is no information on the binding ability of the goby TTX-binding protein(s) to TTX and 5,6,11-trideoxyTTX. It is also suggested that transporters are involved in the uptake of TTX in pufferfish [51].

## 4. Conclusions

The composition of TTX and 5,6,11-trideoxyTTX between the sympatric pufferfish and goby was found to be different in this study. Our results suggest that the TTX-containing prey may be different for the two species, and that the internal mechanism for accumulating ingested TTX and 5,6,11-trideoxyTTX may also be different between the two species. In order to elucidate the toxification mechanism of TTX-bearing organisms, it is necessary to investigate the composition of TTX and 5,6,11-trideoxyTTX in various organisms, as well as the biomolecules involved in their binding, transport, and accumulation. In addition, TTX is highly toxic, whereas 5,6,11-trideoxyTTX is almost non-toxic. Thus, the toxicity of TTXs varies greatly depending on the form of TTX, and the amount of TTX possessed has a great impact on the expression of toxicity. However, 5,6,11-trideoxyTTX is estimated to be a precursor of TTX in the TTX biosynthetic system, and there is a possibility that TTX synthesis was in progress in the prey or in the underlying biota. Promoting such studies may provide an opportunity to identify biota with TTX biosynthetic systems.

## 5. Materials and Methods

### 5.1. TTX-Bearing Fish

The pufferfish (*Chelonodon patoca*) juveniles (15–55 mm TL) were captured in June 2019 from estuarine/coastal waters of Iriomote Island, Japan (“A, 24°18′20′′ N, 123°44′51′′ E” in Figure 1), and Ishigaki Island, Japan (“B, 24°23′30′′ N, 124°08′30′′ E”); in July 2020 from estuarine waters of Okinawa Island, Japan (“E, 26°38′06′′ N, 128°00′05′′ E”; “F, 26°37′57′′ N, 128°01′55′′ E”); and in May and June 2021, from estuarine waters of Ishigaki Island, Japan (“B”). The toxic goby (*Yongeichthys criniger*) juveniles (31–59 mm TL) were captured in June 2019 from estuarine waters of Iriomote Island, Japan (“A”), and Ishigaki Island, Japan (“B” and “C, 24°29′13′′ N, 124°13′50′′ E”); in July 2020 from estuarine waters of Okinawa Island, Japan (“F”); and in May and June 2021 from estuarine waters of Ishigaki Island, Japan (“B”, “C” and “D, 24°32′58′′ N, 124°17′34′′ E”). The sample sizes of both toxic fish species were all *n* = 10 for each sampling time and each region. All fish individuals were anesthetized with ice water after collection, euthanized, and stored at –30 °C until use. Although the research did not require the consent of the ethics committee, all animal procedures complied with the Japanese Government Animal Protection and Management Law (No. 105) and Japanese Government Notification on Feeding and Safekeeping of Animals (No. 6).

### 5.2. LC–MS/MS Analysis

The TTX used in this study was commercially obtained (FUJIFILM Wako Pure Chemicals, Osaka, Japan), while 5,6,11-trideoxyTTX was chemically synthesized [52]. TTX extracts were prepared from the homogenates for the left half the body with skin of the pufferfish and toxic goby, and they were filtered through a membrane of pore-size 0.45 μm (SupraPure Syringe Filter, Recenttec, Taipei, Taiwan). LC–MS experiments were performed on a Shimadzu LC-20AD solvent delivery system and a SCIEX X500R Q-TOF Mass Spectrometer with an ESI ion source. The filtered solution was subjected to LC–MS/MS analysis with multiple reaction monitoring (MRM) using an Atlantis HILIC Silica column (Waters, Milford, MA, USA) with gradient elution, following the method used in a previous study [28]. The optimized transitions for TTX were *m*/*z* 320 > 302 for qualification and *m*/*z* 320 > 162 for quantification, and those for 5,6,11-trideoxyTTX were *m*/*z* 272 > 254 and *m*/*z* 272 > 162. The calibration curve generated with 1–50 ng/mL of TTX standards (TTX and 5,6,11-trideoxyTTX) showed good linearity and precision. The other TTX analogues were not analyzed because they were in very small amounts.

### 5.3. Planocera multitentaculata-Specific PCR Analysis and Sequencing Analysis

Following a previous study [19], a *Planocera multitentaculata*-specific PCR was carried out with some modifications. Total genomic DNA was extracted from the intestinal contents of the pufferfish and goby. The *P. multitentaculata*-specific DNA band encoding partial COI was detected by PCR using the PCR primer set, PMTF1 (5′-TTATT ATTGG GTTCA TTTGT GGTAG AG-3′) and PMTR2 (5′-AATCA TACCA AACCC CGGC-3′) [19], while amplification of a predator-derived DNA band (16S rRNA gene) was performed by PCR using the primer sets 16SAR-L (5′-CGCCT GTTTA TCAAA AACAT-3′) and 16SBR-H (5′-CTGTT BRAAG GGCTT AGGBC TTTTG C-3′) [53], in order to confirm the DNA extraction procedure.

The PCR products were sequenced and compared against the corresponding sequences in the DDBJ/EMBL/GenBank databases. The sequences of the flatworm COI gene were submitted to the DDBJ/EMBL/GenBank databases under the accession numbers LC671559–LC671560.

### 5.4. Statistical Analysis

The statistical significance of differences in the amounts of toxin and related compounds was analyzed by means of a Student’s *t*-test or a one-way ANOVA followed by Tukey’s Honestly Significant Difference (HSD) test. A significance level of 0.05 was set. Data are given as mean ± standard deviation.

## Figures and Tables

**Table 1:** Sampling time and localities, body weight (BW), and concentrations for tetrodotoxin (TTX) and 5,6,11-trideoxyTTX of the pufferfish *Chelonodon patoca* and toxic goby *Yongeichthys criniger* samples used in this study.

Species	Year	Month	Locality	BW (g)	TTX Concentration (μg/g)	TDT Concentration (μg/g)	TTX/TDT Ratio	Sequence of Toxic Flatworm (%)
*Chelonodon patoca*	2019	June	IRI (A) *^1^	0.99 ± 0.48	51.4 ± 16.1	25.0 ± 24.4	3.4 ± 1.4	0
			ISI (B) *^2^	0.94 ± 0.32	30.9 ± 7.4	6.3 ± 2.3	5.6 ± 2.3	0
			OKI (F) *^3^	0.12 ± 0.03	60.4 ± 10.0	37.0 ± 12.0	1.8 ± 0.6	10
	2020	July	OKI (E)	1.13 ± 0.97	6.0 ± 2.2	2.5 ± 1.0	2.4 ± 0.5	70
			OKI (F) *^4^	0.70 ± 0.58	5.2 ± 2.0	1.8 ± 0.8	3.1 ± 1.3	90
	2021	May	ISI (B) *^5^	0.17 ± 0.08	59.8 ± 9.4	16.3 ± 4.3	3.8 ± 0.7	0
		June	ISI (B) *^6^	0.48 ± 0.15	9.8 ± 1.9	2.2 ± 0.3	4.6 ± 1.2	0
*Yongeichthys criniger*	2019	June	IRI (A) *^1^	0.82 ± 0.30	218.7 ± 137.5	270.7 ± 132.2	0.8 ± 0.2	40
			ISI (B) *^2^	0.59 ± 0.26	57.1 ± 16.1	83.9 ± 40.7	0.7 ± 0.2	60
			ISI (C)	0.79 ± 0.31	50.8 ± 35.0	33.6 ± 14.6	1.7 ± 0.8	40
			OKI (F) *^3^	0.70 ± 0.10	226.5 ± 95.4	427.0 ± 56.3	0.5 ± 0.2	0
	2020	July	OKI (F) *^4^	1.35 ± 0.48	99.5 ± 40.3	153.5 ± 54.0	0.6 ± 0.1	70
	2021	May	ISI (B) *^5^	0.34 ± 0.09	98.6 ± 54.7	206.0 ± 52.3	0.5 ± 0.2	0
			ISI (D)	0.52 ± 0.24	185.0 ± 29.7	109.4 ± 38.3	1.9 ± 0.6	0
		June	ISI (B) *^6^	0.61 ± 0.23	40.6 ± 7.6	64.1 ± 27.0	0.7 ± 0.2	0
			ISI (C)	0.60 ± 0.19	107.9 ± 33.1	104.3 ± 36.3	1.1 ± 0.3	0
			ISI (D)	0.96 ± 0.21	192.5 ± 39.1	107.4 ± 20.3	1.8 ± 0.2	0

IRI, ISI and OKI represent Iriomote, Ishigaki and Okinawa Island, respectively. Letters A–F in parentheses represent sampling locations corresponding to waters shown in Figure 1. The sample sizes were all *n* = 10 for each sampling time and each region. BW, body weight; TDT, 5,6,11-trideoxyTTX. Data are presented as mean ± standard deviation in BW, TTX concentration, TDT concentration and TTX/TDT ratio. Student’s *t*-test was employed for statistical comparison between measured values (in the column of TTX and 5,6,11-trideoxyTTX concentration) of the pufferfish and the goby with same superscript (*^1^–*^6^) collected at the same locality (*p* < 0.05). Values under the column “Sequence of toxic flatworm (%)” are the percentages of pufferfish and goby juveniles in which the *Planocera multitentaculata*-specific DNA sequence was detected in their intestinal contents.

## Data Availability

Data are contained within the article.
